# The PITT pathway: Keeping lysosomes young

**DOI:** 10.1002/ctm2.1097

**Published:** 2022-10-25

**Authors:** Haoxiang Yang, Jay Xiaojun Tan

**Affiliations:** ^1^ Aging Institute University of Pittsburgh School of Medicine/University of Pittsburgh Medical Center Pittsburgh Pennsylvania USA; ^2^ Department of Cell Biology University of Pittsburgh School of Medicine Pittsburgh Pennsylvania USA

1

Lysosomes are essential subcellular organelles in animal cells discovered by Christian de Duve in 1950s. Carrying many hydrolytic enzymes, lysosomes not only control nutrient recycling and cellular growth, but also mediate the proper handling of various cellular stressors, including the clearance of pathogens and damaged macromolecules like protein aggregates.^1^ With many important functions dependent on one type of organelle, it creates the risk of developing diseases when lysosomes are compromised. Lysosomal dysfunction is known to be associated with aging and many diseases such as neurodegenerative and cardiovascular diseases, usually exhibited as expansion of compromised lysosomes with reduced hydrolytic activity.^2^

A hallmark of lysosomal‐related diseases is lysosomal membrane permeabilization (LMP). Given the detrimental consequences of impaired lysosomal integrity and the high frequency of LMP in diseases and normal aging,^2^ animal cells must have evolved essential mechanisms to rapidly repair damaged lysosomes. Three distinct lysosomal quality control pathways have been previously reported (Figure 1) including (1) the transcription factor EB (TFEB) pathway that upregulates lysosomal biogenesis in response to lysosomal damage,^3^ (2) lysophagy as an indirect lysosomal repair mechanism that removes damaged lysosomes through autophagy,^4^, ^5^, ^6^ and (3) the endosomal sorting complex required for transport (ESCRT) pathway for direct and rapid repair of small lysosomal membrane pores.^7^, ^8^, ^9^ While TFEB and lysophagy are important in replacing damaged lysosomes with new ones, they likely are unable to respond rapidly enough to block acute lysosomal leakage and thus would not effectively protect cells from lysosomal cell death signaling.^2^ ESCRT would be an appealing candidate to block lysosomal leakage through direct membrane repair. However, despite rapid ESCRT recruitment to damaged lysosomes, its depletion does not substantially delay rapid lysosomal repair.^7^, ^8^, ^9^ Thus, additional lysosomal repair mechanisms exist in animal cells.

**FIGURE 1:**
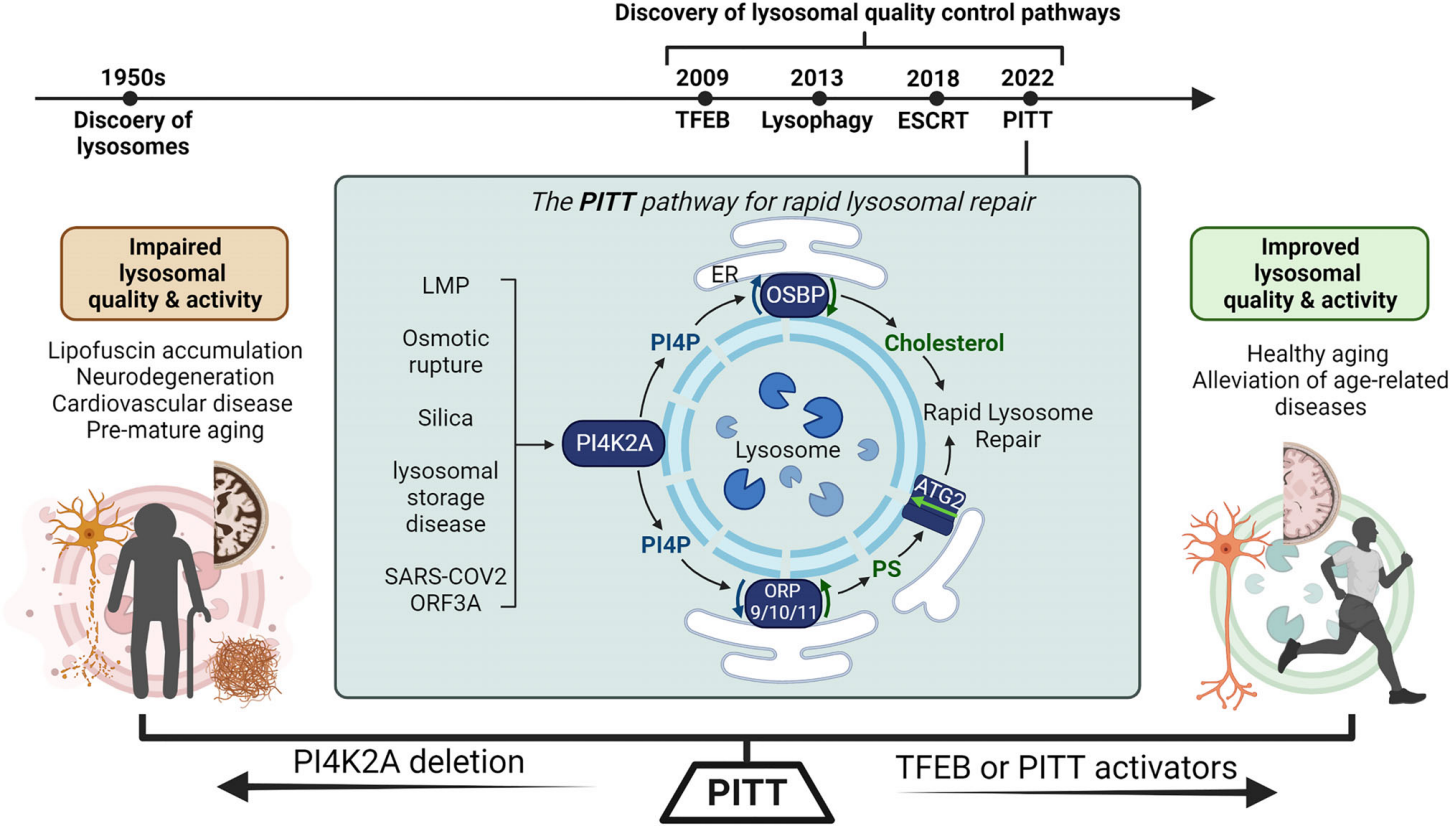
Fixing old lysosomes: The PITT pathway to the rescue. The phosphoinositide‐initiated membrane tethering and lipid transport (PITT) pathway is essential for rapid lysosomal repair. It is activated by multiple distinct, disease‐related lysosome‐damaging conditions, suggesting that it is a common mechanism for lysosomal quality control. The PITT pathway starts with robust lysosomal recruitment of PI4K2A, an enzyme that produces large amounts of the lipid messenger phosphatidylinositol‐4‐phosphate (PI4P) on damaged lysosomes. PI4P drives the formation of extensive, new membrane contact sites (MSCs) between the endoplasmic reticulum (ER) and damaged lysosomes by recruiting four oxysterol‐binding protein (OSBP)‐related protein (ORP) family members. OSBP and ORPs subsequently exchange lysosomal PI4P into cholesterol and phosphatidylserine (PS), respectively. Cholesterol by itself protects lysosomal membrane, and lysosomal PS activates autophagy protein 2 (ATG2)‐mediated lipid transfer for direct membrane repair. Loss of the PITT pathway due to deletion of PI4K2A is known to cause neurodegeneration and pre‐mature aging in both mice and human patients. TFEB or PITT activators are expected to improve lysosomal quality and activity, and thus may benefit human health, promoting healthy aging and alleviating age‐ and lysosome‐related diseases. Figure created with BioRender.com

To identify additional mechanisms for rapid lysosomal repair, we designed an unbiased proteomic screen to search for proteins specifically enriched on damaged lysosomes. Through proximity labelling, we purified all lysosomal surface proteins before and after lysosomal membrane damage for mass spectrometry analysis. This approach led to our recent discovery of the phosphoinositide‐initiated membrane tethering and lipid transport (PITT) pathway that is essential for rapid lysosomal membrane repair (Figure 1).^10^ The PITT pathway is quickly triggered by LMP and it relies on new de novo membrane contacts between two organelles, namely damaged lysosomes and the endoplasmic reticulum (ER). In particular, we found that LMP stimulates rapid lysosomal recruitment of type II alpha phosphatidylinositol‐4 kinase (PI4K2A), leading to the production of phosphatidylinositol‐4‐phosphate (PtdIns4P, PI4P) on damaged lysosomes.^10^ PI4P drives the formation of extensive ER‐lysosomal membrane contacts by recruiting multiple oxysterol‐binding protein (OSBP)‐related protein (ORP) family members, including ORP9, ORP10, ORP11, and OSBP.^10^ Loss of either PI4K2A or the ORP proteins dramatically delays rapid lysosomal repair.^10^

The ORP proteins are not only membrane tethers between the ER and damaged lysosomes, but also lipid transporters catalyzing ER‐to‐lysosome transfer of cholesterol and phosphatidylserine (PS).^10^ The four ORPs in the PITT pathway fall into two groups (Figure 1). ORP9, ORP10, and ORP11 form heterodimers and mediate the PI4P/PS counter transport at ER‐lysosome membrane contacts, whereas OSBP form homodimers for PI4P/cholesterol exchange.^10^ Interestingly, lysosomal accumulation of either cholesterol or PS appears sufficient to support rapid lysosomal repair.^10^ Of note, the role for PI4K2A in rapid lysosomal repair and the accumulation of cholesterol and PS on damaged lysosomes have also been observed in another recent study, which focused on the impact of cholesterol on lysosomal membrane stabilization.^11^

While cholesterol by itself protects lysosomal membrane, we found that lysosomal PS activates autophagy protein 2 (ATG2)‐mediated lipid delivery for direct lysosomal repair^10^ (Figure 1). This new function of ATG2 is completely independent of its well‐established role in supplying lipids for autophagosome formation. Although in vitro evidence for robust lipid transfer by ATG2 is still missing, the roles for ATG2 in autophagy and rapid lysosomal repair are strong indications of high speed, large‐scale lipid transfer. Little was known previously about lipid changes in lysosomal stress response. Now the PITT pathway establishes lipid remodelling as a new platform to understand lysosomal quality control mechanisms. Robust lipid remodelling here is exemplified by the triggered PI4P lipid signalling, the ER‐to‐lysosome transfer of cholesterol and PS, and the larger scale lysosomal lipid delivery by ATG2. There are likely additional lipid remodelling events in lysosomal quality control.^12^

The PITT pathway is triggered by PI4P signalling that drives extensive ER‐lysosome membrane contacts to support multiple lipid transfer processes for direct and rapid lysosomal repair. The lysosomal recruitment of the first enzyme of the PITT pathway, PI4K2A, is activated by multiple exogenous agents or disease‐related conditions that damage lysosomal membrane through distinct mechanisms^10^ (Figure 1). For instance, it is activated by various chemicals that trigger LMP or osmotic rupture, by silica which causes silicosis, a long‐term lung disease, and by gene editing that recapitulates a known lysosomal storage disease. Thus, the PITT pathway appears to be a commonly invoked mechanism for rapid lysosomal repair.

The PITT rapid lysosomal repair pathway is expected to have significant impact on human physiology and pathology, especially for post‐mitotic cells, such as neurons and cardiomyocytes that rely heavily on lysosomal activity for homeostasis. For instance, we found that in cellular models loss of the PITT pathway exacerbated tau fibril spreading,^10^ a key step in the progression of Alzheimer's disease that relies on lysosomal membrane damage by endocytosed tau fibrils.^13^ Loss of the PITT pathway also increases cellular accumulation of lipofuscin,^10^ a pathological finding in old lysosomes and a known hallmark of aging. In mouse models and human patients, loss of the first enzyme of the PITT pathway causes severe neurodegeneration and pre‐mature aging.^14^, ^15^, ^16^ The PITT pathway is therefore a new insight toward understanding how lysosomal dysfunction contributes to aging and disease.

Multiple long‐lived animal models are able to maintain lysosomal function as they age, while human genetics suggest that lysosomal dysfunction can contribute to a host of age‐related diseases.^17^ Indeed, improved lysosomal quality and activity is connected to most validated anti‐aging interventions that extend health‐span or life‐span of model organisms.^17^ It is expected that activation of key proteins in the PITT pathway by small molecules may increase lysosomal activity, delay or alleviate aging and age‐related diseases. We are now searching for small molecule PITT activators to explore potential health benefits of activating the PITT pathway in relevant disease models.

## CONFLICT OF INTEREST

The authors declare no conflict of interest.
